# Galactosemia: Towards Pharmacological Chaperones

**DOI:** 10.3390/jpm11020106

**Published:** 2021-02-07

**Authors:** Samantha Banford, Thomas J. McCorvie, Angel L. Pey, David J. Timson

**Affiliations:** 1 South Eastern Health and Social Care Trust, Downpatrick BT30 6RL, UK; Samantha.banford@nhs.net; 2 Structural Genomics Consortium, University of Oxford, Oxford OX3 7DQ, UK; thomas.mccorvie@sgc.ox.ac.uk; 3 Departamento de Química Física, Unidad de Excelencia de Química aplicada a Biomedicina y Medioambiente e Instituto de Biotecnología, Facultad de Ciencias, Universidad de Granada, 18071 Granada, Spain; angelpey@ugr.es; 4 School of Pharmacy and Biomolecular Sciences, The University of Brighton, Brighton BN2 4GJ, UK

**Keywords:** galactose metabolism, enzyme, protein misfolding, protein degradation, ligand binding, galactose 1-phosphate uridylyltransferase, galactokinase, UDP-galactose 4′-epimerase, galactose mutarotase, drug screening

## Abstract

Galactosemia is a rare inherited metabolic disease resulting from mutations in the four genes which encode enzymes involved in the metabolism of galactose. The current therapy, the removal of galactose from the diet, is inadequate. Consequently, many patients suffer lifelong physical and cognitive disability. The phenotype varies from almost asymptomatic to life-threatening disability. The fundamental biochemical cause of the disease is a decrease in enzymatic activity due to failure of the affected protein to fold and/or function correctly. Many novel therapies have been proposed for the treatment of galactosemia. Often, these are designed to treat the symptoms and not the fundamental cause. Pharmacological chaperones (PC) (small molecules which correct the folding of misfolded proteins) represent an exciting potential therapy for galactosemia. In theory, they would restore enzyme function, thus preventing downstream pathological consequences. In practice, no PCs have been identified for potential application in galactosemia. Here, we review the biochemical basis of the disease, identify opportunities for the application of PCs and describe how these might be discovered. We will conclude by considering some of the clinical issues which will affect the future use of PCs in the treatment of galactosemia.

## 1. Introduction

**Figure 1:**
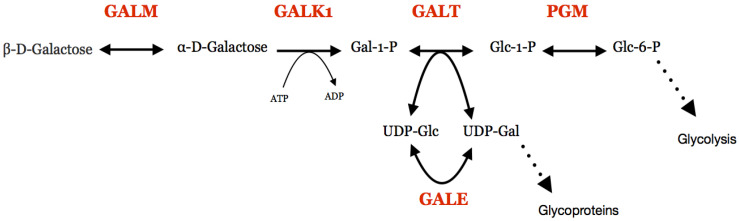
The Leloir pathway and associated reactions. Protein names are shown in red. GALM, galactose mutarotase; GALK1, galactokinase; GALT, galactose 1-phosphate uridylyltransferase; PGM, Phosphoglucomutase; GALE, UDP -galactose 4′-epimerase; Gal, galactose; Glc, glucose.

Galactosemia is an inherited metabolic disease which causes deficiency in the metabolism of galactose and in the formation of galactose containing products in the body [1,2,3,4,5,6]. In normal metabolism, the Leloir pathway takes galactose and converts it into glucose 1-phosphate (Figure 1). This product can be further metabolized to glucose 6-phosphate, which enters glycolysis for energy production in the cell. The Leloir pathway has four steps, each catalysed by a different enzyme [7,8,9]. The types of galactosemia are segregated depending on which enzyme the deficiency is present in [2].

Clinical manifestations of the disease include cataracts, low IQ, neonatal jaundice, infantile failure to thrive, *Escherichia coli* sepsis, neuromuscular dysfunction, infertility or miscarriage [10,11,12,13,14]. Most severe symptoms occur in early life when children are dependent on milk for nutrition. The severity of symptoms depends on which enzyme is affected, the mutation’s effect on the protein and the patient’s environment, including diet. Some of the effects of the condition can be prevented by the avoidance of galactose containing foods in the diet, which should start as soon as possible after birth [11,15]. 

Type I galactosemia (OMIM#230400), also known as classic galactosemia, is the most commonly diagnosed, and often has the most severe symptoms. It is caused by a mutation in the GALT gene which codes for the enzyme galactose 1-phosphate uridylyltransferase (GALT; EC 2.7.7.12) [16,17]. p.Q188R is the most common mutation in Caucasians, manifesting with life-threatening symptoms in the neonatal period [18,19]. In the black population, the p.S135L variant is more commonly detected. Phenotypically, it is associated with slightly better clinical outcomes than p.Q188R [20,21,22]. Type II galactosemia (previously known as galactokinase deficiency; OMIM#230200) is caused by mutations in the gene coding for the enzyme galactokinase (GALK1; EC 2.7.1.6) [23,24]. It is rarer than type I, and often less severe, typically manifesting in early onset cataracts [25]. Type III galactosemia (OMIM#230350) is caused by mutations in the *GALE* gene, which codes for the enzyme UDP-galactose 4′-epimerase (GALE; EC 5.1.3.2) [26]. It usually has the mildest symptoms. In some forms, it is thought the disease only affects red blood cells. The most severe forms are phenotypically similar to type I galactosemia [27,28]. This enzyme is not only critical for the use of galactose as an energy source, but also in the process of attaching galactose sidechains to other proteins, carbohydrates and lipids [29]. Type IV galactosemia (OMIM#137030) is caused by mutations in the *GALM* gene causing deficiency in the galactose mutarotase enzyme (GALM; EC 5.1.3.3) [30,31,32,33]. It has only been recently discovered that mutations in this gene lead to a disease state. It presents with symptoms similar to type II [31].

**Figure 2:**
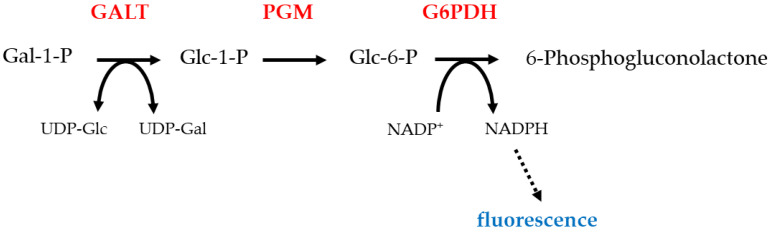
Beulter test: this test detects the fluorescence produced by the NADPH. NADPH is made by the action of galactose 6-phosphate dehydrogenase on glucose 6-phosphate [34]. The absence of fluorescence, which would occur in GALT deficiency, is a positive test. Protein names are shown in redGALT, galactose 1-phosphate uridylyltransferase; PGM, Phosphoglucomutase; G6PDH, glucose-6-phosphate dehydrogenase; Gal, galactose; Glc, glucose.

The most commonly used diagnostic test for galactosemia is the Beulter test (Figure 2) [34]. This relies on detecting the conversion of galactose 1-phosphate to gluconate 6-phosphate, a process which requires the activities of GALT, phosphoglucomutase (PGM) and glucose 6-phosphate dehydrogenase (G6PDH). The last step uses NADP^+^ as an oxidizing agent. The reduced cofactor, NADPH, can be detected by fluorescence. Thus, a positive result for type I galactosemia is the absence of this fluorescence. False positives result from blood samples which contain EDTA, samples exposed to heat, and deficiency of PGM or G6PDH [35]. An alternative test uses a non-specific phosphatase enzyme to convert galactose 1-phosphate to galactose. Galactose is then detected by oxidation and catalysed by galactose dehydrogenase, an enzyme which does not occur in humans. This requires NAD^+^ as a cofactor, and the presence of the reduced form indicates a positive result and is detected by fluorescence [36]. Tandem mass spectrometry is also a widely used method to determine both galactose 1-phosphate [37] concentration and GALT activity [38]. Galactose 1-phosphate concentrations are raised in the blood of type I (and sometimes type III) galactosemia patients. While concentrations of this metabolite are not raised in type II galactosemia, galactose concentrations are increased and so false positives and consequent misdiagnosis can occur. Diagnosis is normally confirmed by molecular biology methods. Historically, these methods involved the use of site-specific probes to identify common mutations, which inevitably missed rarer and novel mutations. Therefore, complete sequencing of the suspected causative gene is recommended to ensure a personalized medicine approach. Some countries include galactosemia in newborn screening programs [39]. In many cases, only GALT deficiency is tested for since this is the most common and severe form, but lower rates of testing for the other types could be one reason why they are less commonly diagnosed. It is also possible that types II, III and IV can result in very mild or almost asymptomatic phenotypes which remain undiagnosed. For example, the p.A198V variant of GALK1 results in an increased risk of cataracts in mid and later life [40]. A further useful test is measuring urine galactitol, which can be performed by GC/MS or gas chromatography [41]. Enzyme levels can also be determined through enzymatic assays.

Attempts have been made to try and elucidate how the genotype of galactosemia links to the phenotype expressed in patients by using yeast, mouse, rat, nematode, zebra fish and fly models [42,43,44,45,46,47,48,49,50,51,52,53,54]. However, significant questions are still unanswered. Mechanisms such as the build-up of toxic metabolites (i.e., galactose, galactose 1-phosphate or galactitol) have been thought to explain some but not all symptoms [55]. How these increased metabolite levels result in complex pathology affecting multiple organ systems is contentious. 

Apart from the avoidance of galactose containing food products, no other specific treatments are commonly used in clinical practice. Other treatments used are symptom specific, such as fertility treatments for female patients [14]. The total elimination of galactose is impossible as some galactose is made in vivo, and some is required in the making of galactose containing products (in type III only). Therefore, long-term side-effects are inevitable [56,57,58]. Treatments under investigation include GALK1 inhibitors, antioxidants, aldose reductase inhibitors, gene therapy, mRNA therapy, stress signalling pathway inhibitors and enzyme replacement therapy (recently reviewed in [59,60,61,62,63]). Another potential strategy is the use of pharmacological chaperones (PCs). This strategy works by using a small molecule to bind to the affected protein to increase its stability, as many of the disease-causing mutations cause protein instability [64,65,66,67]. The chaperone may also aid in preventing protein misfolding (see below).

## 2. Pharmacological Chaperones

PCs are a potential therapeutic approach for protein misfolding and aggregation diseases [68]. PCs consist of small molecules that bind to the target proteins, stabilising disease-associated variants [69,70]. Their preferential binding to the native/folded state prevents the conformational fluctuations from destabilising the target protein, hindering misfolding and aggregation. This then shifts the equilibrium towards the folded form, resulting in a higher amount of protein that avoids the quality control machinery, decreases aberrant trafficking, and ultimately increases the active pool of enzyme. As such, PCs have been proposed to correct the folding and trafficking defects for not only enzymes, but transporters, receptors, and other structural proteins as well [68]. Like other small molecule drugs, PCs have a number of potential advantages over other treatments, such as oral delivery, broad biodistribution, and lower costs. They can be less burdensome to patients. Importantly, their ability to cross the blood–brain barrier to reach different target tissues can allow for the treatment of neuropathic pathologies present in many rare disorders. It has been proposed that increasing protein function to moderate levels beyond a threshold (e.g., 10%) would be sufficient to delay the onset and slow the clinical progression of these disorders [68]. Notably, though suggested to target stability and misfolding issues of disease-associated variants, PCs can likewise stabilise and increase levels of the wild type protein, which can increase the efficacy of enzyme replacement therapies (ERT) if co-administered [71]. For a small molecule to act as a PC, it must have a (reasonably) high binding affinity and specificity for the wild type or disease variant of the protein, higher in the folded than in partially folded state(s). The binding affinity of the PC molecule generally correlates with its potential to rescue misfolding and aggregation. Fundamentally, native ligands of target enzymes, such as substrates, cofactors, and products with well-known interactions, can serve as templates for the development of PCs [72]. Therefore, early-stage proof-of-concept and discovery studies have relied heavily upon protein structural information, allowing for in-depth analysis at the molecular level of the effects of disease associated mutations and characterising protein-small molecule interactions for drug design.

For misfolding disorders, a number of PC therapies have reached the clinical setting, each with their own specific mode of action serving as paradigms for efforts in other disorders. An example of a substrate mimetic PC can be found in the development for the treatment of Fabry disease (OMIM #301500), an X-linked disorder that reduces the activity of α-galactosidase A (GLA). This disease is representative of lysosomal storage disorders (LSDs) [73]. LSDs are a group of inherited diseases characterized by toxic accumulation of glycosphingolipids, glycoproteins or mucopolysaccharides in patient lysosomes. The iminosugar 1-deoxygalactonojirimycin (DGJ, migalastat hydrochloride) mimics the terminal galactose of GLA’s native substrate and has been shown to increase GLA activity in cultured cells, mouse models, and clinical trials [74], leading to the approval of the drug by the European Union in 2016 (Galafold) [74]. 

Another example is cystic fibrosis (CF, OMIM #219700). CF is caused by defects in the ATP-binding cassette -type chloride channel CFTR (cystic fibrosis transmembrane regulator). Commonly, mutations result in protein misfolding and mis-trafficking, which leads to proteasome degradation [75]. Consequently, there have been intensive efforts aimed at developing PCs that either increase channel activity (“potentiator”) or improve CFTR trafficking (“corrector”) [76]. These efforts have resulted in the development of Ivacaftor, an FDA-approved potentiator. Invacaftor increases channel activity and lung function in CF patients with the p.G551D variant. Lumacaftor/Tezacaftor are correctors that improve the folding and increase trafficking of the p.ΔF508 variant, which is the most common mutation for CF [77]. 

A PC therapy for hereditary transthyretin (TTR)-related amyloidosis (OMIM #105210) has also been developed. This disorder is an autosomal dominant disease of progressive neuropathies and cardiopathies caused by mutations in *TTR*. TTR is a transporter for thyroxine and holo retinol-binding protein. Disease-related mutations tend to cause TTR destabilization, leading to the native tetramer disassociating into monomers that misfold and trigger amyloid aggregation [78]. Therefore, a PC approach that stabilizes the tetrameric form, reducing the build-up of the amyloidogenic monomer and preventing TTR aggregation, was developed: Tafamidis, a benzoxazole derivative which has been shown to slow disease progression for certain genotypes of the disorder [79]. Significantly, the crystal structure reveals how this molecule occupies the two thyroxine-binding sites that are usually unoccupied under physiological conditions [80]. Binding causes the stabilization of the dimer–dimer interface and provides a structural basis for kinetic stabilization. This reduces the tetramer-to-monomer disassociation and thus prevents amyloidogenesis.

## 3. Potential in the Treatment of Galactosemia 

The fundamental biochemical cause of galactosemia is often failure of the affected protein to fold correctly and/or its instability. This has been demonstrated in all four types of the disease. Some variant forms of GALT have been shown to be more sensitive to protease digestion and thermal denaturation than the wild type, thus supporting mutational effects on local and global stabilities of the enzymes [81,82,83]. Although only a minority of variants have been tested, computational studies suggest conformational instability may be a general effect for the majority of disease associated variants [84,85]. However, it should be noted that some disease associated variants (e.g., p.D28Y, p.L74P and p.F171S) are more stable against proteolysis and thermal denaturation, suggesting that some degree of flexibility is required for efficient catalysis and regulation [81]. Variants of galactokinase which are associated with the most severe forms are insoluble on expression in *E. coli* [86,87]. This suggests that they failed to fold and aggregated. The prokaryotic cellular environment is different to its eukaryotic equivalents, and so some caution is required in generalising this conclusion to human cells. Nevertheless, it is likely these variants fold less efficiently than the wild type. Some variants, including the one associated with the mildest known form of type II galactosemia (p.A198V), are expressed in reduced amounts by cells in comparison to the wild type [40,88]. Computational studies support the hypothesis that protein instability is a key factor in causing type II galactosemia [89,90]. Disease associated variants of UDP-galactose 4′ epimerase are typically less stable then the wild type and, in some cases, have reduced affinity for the NAD^+^ cofactor or aggregate in vivo [91,92,93,94,95,96]. Experimental and computational studies suggest there is a correlation between the degree of misfolding and the severity of the associated disease [92,97,98]. Similarly, in type IV galactosemia, experimental and computational studies link protein instability to reduced enzymatic activity and disease [30,31]. 

Taken together, these in silico, in vitro and in vivo data strongly suggest that misfolding is the main molecular mechanism in the pathology of galactosemia. Therefore, PCs may be an attractive therapy since they would help correct misfolding. However, the relatively mild symptoms normally associated with types II and IV, along with some forms of type III, may make it unrealistic to presume a PC strategy in these cases. The rarity of severe cases of type III may make the development of chaperones for this condition commercially unviable. Thus, type I represents the most likely form of the disease for which commercially and clinically sound PC strategy may be credible. Even in this type, the diversity of mutations may mean that PC treatments are only possible for a subset of variants. In particular, those variants with increased rigidity would be poor prospects for correction by chaperones. While protein misfolding is the most common fundamental cause of this disease, there are others, for example, mutations which directly affect the active site residues and prevent catalysis. Such variants would also not be addressed by a PC. The most common variants, p.S135L, Q188R, and K285N, in GALT are destabilised, and therefore represent viable targets for treatment [82,83]. Ideally, one molecule would be identified which corrects the folding of as many different disease-causing variants as possible. In Q188R, the most common variant in Caucasians, the mutation not only distorts the overall protein but severely distorts the active site [82,99]. The PC would need to correct this.

Initial studies suggested that non-specific binding of the amino acid arginine stabilises GALT [100]. Unfortunately, further work demonstrated that this is not the case [101]. In contrast, PCs bind to specific sites. This requires the identification of sites which contain sufficient exposed functional groups to allow respectable binding affinity and specificity. At least two such sites are potentially useful in human GALT. First, there are the two active sites. While it may seem counter intuitive to develop specific active site binding molecules since they are almost certain to act as competitive inhibitors, there are established cases of PC-like-molecules which bind to active sites (e.g., [102]). In the case of GALT, it was established experimentally that binding to the substrates increases the thermal stability of the protein [81]. Given that GALT has two active sites, if one site is occupied by a stabilising molecule (with affinity in the range of the natural substrate/coenzyme), this would stabilise the overall assembly and increase the catalytic efficiency of the second active site. The second potential chaperone binding site was identified computationally in a model of the enzyme, and its existence was confirmed in the crystal structure [81,82]. This pocket may act as a binding site for allosteric modifiers of the enzyme, but no such modifiers have been identified. GALT demonstrates cooperativity towards its substrates under some conditions in vitro, suggesting the possibility of allosteric communication within the enzyme [81]. 

## 4. Towards the Discovery of Pharmacological Chaperones for Galactosemia 

**Figure 3:**
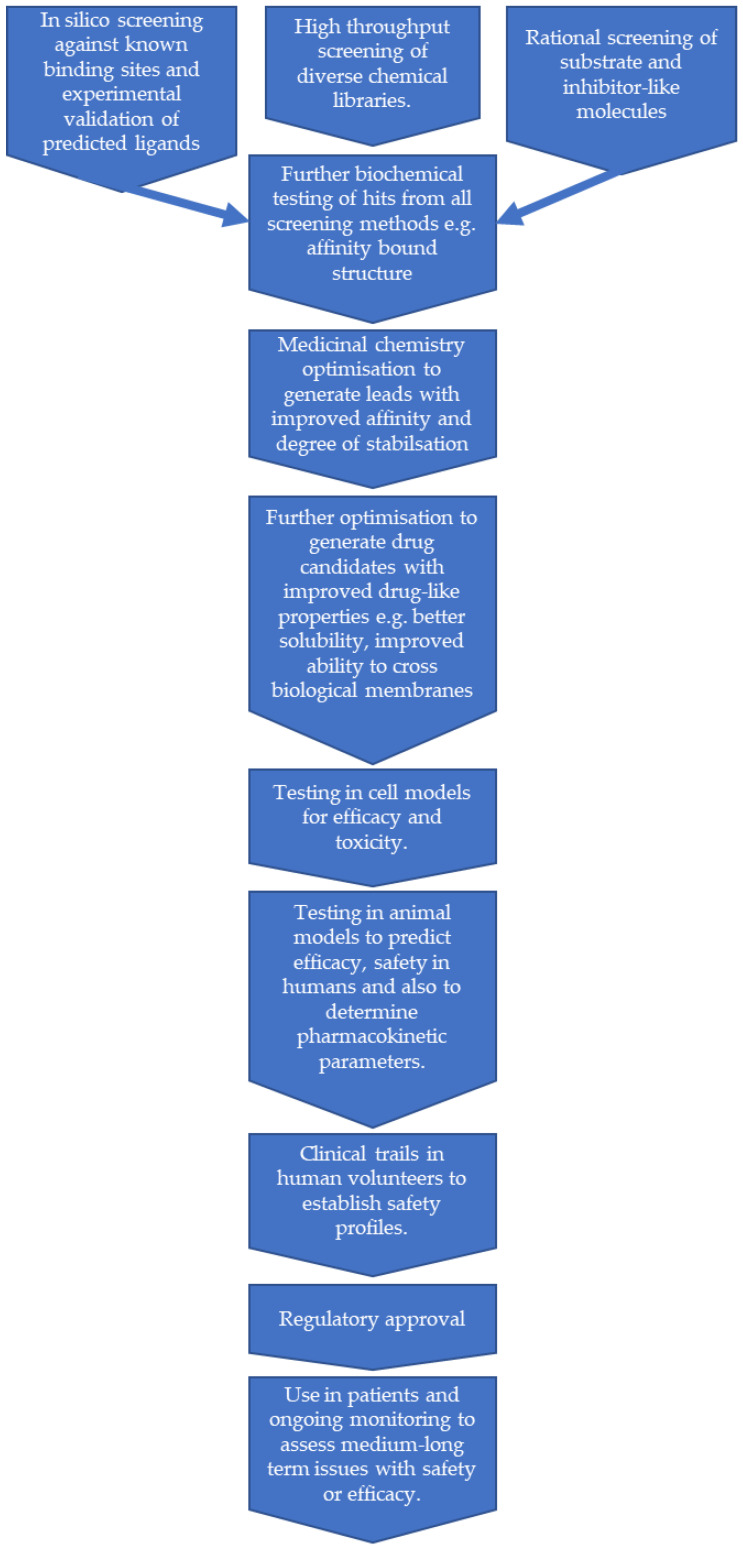
A flow chart showing a possible route for pharmacological chaperones for galactosemia. This represents an idealised view, in reality some steps may need to be repeated and others performed in parallel.

The procedures to identify lead compounds as potential PCs can be widely classified in two groups: experimental and computational-structure guided screenings [103,104,105,106]. The possible route to finding PCs in galactosemia is illustrated in Figure 3.

### 4.1. Experimental Approaches

Experimental procedures require a setup for high-throughput screening that uses a suitable probe for the interaction between the target protein and the ligand. In this group, we can find different approaches depending on the physical and biological properties of the target protein (e.g., soluble vs. membrane protein, folded vs. natively unfolded protein, protein with enzymatic activity and/or allosteric regulation). 

A general and classical approach for soluble proteins, either folded or natively unfolded proteins, is the thermal up-shift assay (more recently, this approach has been developed for membrane proteins) [105,107]. The rationale for this approach is straightforward: preferential binding of a ligand to the native state of the protein (i.e., with higher affinity for the native state) will increase its stability in comparison to those of non-native states. Note that this premise universally holds for equilibrium unfolding. However, it can also be extended in many cases for irreversibly denaturing proteins, as long as ligand binding provides significant kinetic stabilization upon binding to the folded state [108,109,110]. Therefore, it is relatively straightforward to develop high-throughput screening assays in which hits are identified by the increasing thermal stability of the protein, allowing for experimental uncertainty. This approach has been successfully applied to many protein targets in recent years [111,112,113]. Since the hits result in the stabilization of the protein in vitro, we can normally expect that this stabilization will translate into stabilization inside the cell, preventing protein aggregation or degradation of misfolded variants. This is reasonable because the destabilizing effect of mutations required for triggering protein degradation is rather low (about 8–12 kJ·mol^−1^), well within the achievable ligand-induced stabilization [114,115,116,117,118,119]. In other cases, protein degradation may depend on local destabilization of the protein structure, which initiates degradation, rather than in global destabilization [120,121,122,123]. Consequently, a disease-associated variant may bind and become globally (i.e. thermally) stabilized upon ligand binding, but this binding event may not affect the initiation site for degradation and thus will not be able to rescue the activity [123,124]. The relationships between global and local stability dynamics resulting in loss-of-function mechanisms in galactosemias are, to the best of our knowledge, not well-known. However, enhanced sampling of non-native, dynamic and partially unfolded conformations has been described for GALE disease-associated variants. These lead to reduced catalytic performance, and these fluctuations are modulated by native-state ligands such as NAD^+^ [125,126]. We may anticipate that the enhanced fluctuations in the native state due to disease-associated variants could also hold for degradation-prone variants. Thus, the identification of novel lead compounds (particularly those not binding to active site, or allosteric ligands) could be of pharmacological use to treat GALE deficiency [126] as well as other galactosemias. 

A second approach to identify hits for the treatment of galactosemias is enzymatic assays. Like the thermal up-shift assays, these can be readily setup for high-throughput screening. Either ligands that inhibit the activity (but stabilize the enzyme) or those that restore the activity of variants by acting as allosteric chaperones could be identified. 

A third group of assays are those based on screening in eukaryotic cells [106]. These assays are potentially more problematic since handling eukaryotic cells is usually more challenging than working with purified proteins. However, several good models of galactosemia-causing variants have been developed in budding yeast (*S. cerevisiae*). These allow the straightforward measurement of enzyme activity and protein levels, which are related to protein stability [81,91,94]. Thus, these models represent an excellent starting point to develop high-throughput screens [127,128].

An alternative method to high throughput screening is the fragment-based approach [129,130]. This requires protein crystals which are soaked with small organic molecules and the resulting structure solved. The organic molecules are chosen for their similarity to common building blocks (e.g., acetane, phenol) present in more complex molecules. Although these small molecules typically bind with low affinity, superimposition of the structures with different small molecules bound enables the prediction of more complex molecules. These are more likely to bind with high affinity and specificity. Further improvements in both of these attributes can be achieved through conventional medicinal chemistry.

### 4.2. Computational Approaches

Computational approaches can be used to help identify possible binding sites and small molecules which may bind to these. This is achieved by using the protein’s three-dimensional structure to screen potential small molecules. In theory, this process should be similar to screening for inhibitors or antagonists, which is routinely undertaken in drug discovery, but there are two main differences. First, in conventional drug discovery, the binding site is often already known (e.g., the active site of an enzyme or the ligand binding site of a receptor). Second, in the discovery of PCs, we aim to improve or restore function, whereas conventional drug discovery typically seeks to inhibit or block function. To address the first problem, there are programs which can identify possible binding pockets on the surface of proteins (see above for an example of this applied to GALT). Once a potential pocket has been identified, molecules can be screened using conventional methods. This will result in a prediction of molecules which will bind at the site ranked by estimated affinity. While many protein–ligand interactions result in the stabilization of the protein, it is not necessarily the highest affinity binders which will result in the greatest stabilization. Some insight into the degree of stabilization might be obtained from molecular dynamics simulations, but these are time consuming and it may be preferable to move directly to experimental measurements of stabilization. To the best of our knowledge, no computational screening for potential PCs has been carried out with any of the enzymes associated with galactosemia.

## 5. Potential Issues with Clinical Use

Ideally, the PC for type 1 galactosemia would be administered as soon as possible after birth. In the absence of pre-birth diagnosis, this is likely to be after the baby’s first milk feed. Rapid testing would then be required to establish a diagnosis, followed by sequencing of the GALT gene. If the drug had few side effects, then it might be given as soon as galactosemia was suspected. It could be discontinued in the event of a different diagnosis or if gene sequencing revealed the patient had a mutation which was unsuitable for the drug. In reality, the situation is likely to be more complicated, and there are some potential issues with the long-term use of PCs in this disease.

### 5.1. Screening, Testing and Sequencing

Pre-natal screening is routinely performed for a number of genetic conditions (e.g., cystic fibrosis) if a strong family history is present [84]. This would be ideal to allow the most rapid treatment, but this type of testing can be invasive (e.g., amniocentesis). It also presents ethical issues. This topic also raises the question as to when the phenotypic features of the disease first develop. While it is assumed that the majority of the disease processes occur postnatally, this is not known. Galactose, galactose 1-phosphate and galactitol have all been detected in the liver and amniotic fluid of foetuses at 20 weeks gestation. In some rare cases, newborns have been found to have cataracts, which would suggest some pathology can occur in utero [131,132,133]. Some aberrant glycosylation may occur in the foetus. This may be particularly harmful if it occurs in the brain or nervous system. Clearly, if PC strategy relies on being able to protect a healthy newborn, the benefits may be reduced if significant damage has been done in utero. If a prenatal diagnosis was made, there is the potential that in utero treatment with a PC could be commenced.

Newborn screening is performed in many countries for galactosemia. The timing of these tests varies between 24 h to several weeks post birth. In the most severe cases, the baby would have presented with severe symptoms before results are available. This type of screening may be most useful in detecting the milder phenotypes [36]. Delays occur in the testing and sequencing of potential galactosemia sufferers due to lack of capacity in many healthcare systems. Often, samples need to be shipped to tertiary sites for testing and results must be analysed before being sent back. All of this takes time and would delay treatment [85].

With over 300 mutations known to cause type 1 galactosemia, it is unlikely that the molecular mechanism resulting in disease will be identical in all cases. Experimental and computational evidence suggests that the majority of point mutations result in protein misfolding and, therefore, may be amenable to correction by PCs (see Section 3). However, mutations which affect active site residues, cause deletions, or cause defects in mRNA splicing will not be corrected by a PC. Some point mutations may also result in proteins which are refractory to having their structures restored by PCs, e.g., mutations which affect residues in the drug binding site result in increased rigidification of the protein or result in a protein which is so destabilised that the chaperone is insufficient to restore function. Sequencing will be vital to determine if the patient has at least one mutation which is amenable to the action of PCs.

### 5.2. Dosing

With most drugs, adult dosages are straightforward. However, as this drug would be used soon after birth, achieving a therapeutic and non-toxic dose will be difficult to ascertain, but this is important to its effectiveness. The drug dosage will need to be tailored to weight, age and/or body surface area and adjusted as the patient grows. It may also be necessary to tailor the dose to the individual patient’s response as metabolism will vary between individuals, as people mature at different rates. Thus, doses may need to be personalised and are likely to vary until the patient reaches adulthood or beyond. There is some evidence that the dietary restriction of galactose can be relaxed in adult patients [86]. Therefore, a reduced dose may be effective in some adult patients. Ideally, the effectiveness of the dose would be monitored and adjusted according to blood galactose 1-phosphate levels. Other biomarkers such as the incorporation of galactose into glycoproteins may also be useful [87]. Given the risk of toxicity (see below), it may be necessary to check for effects on liver and kidney function (serum urea and electrolytes and liver function tests).

Initially, the only source of dosing information will be from clinical trials, which are likely to have relatively small numbers of patients enrolled due to the rarity of the disease. As with all potential drugs for use in children, clinical trials are very challenging due to ethical approvals and challenges with recruitment. It would be difficult to extrapolate doses for all children at different maturities based on very limited trial data. As a consequence, it will be important that post trial data on dosing in initial recipients are shared effectively. The existence of patient registries such as GalNet may help keep in contact with post trial patients for longer [88].

### 5.3. Toxicity

All drugs have side effects. These can arise through interaction with non-target biomolecules, toxic metabolites or unwanted effects resulting from interaction with the target. Since the PC would increase GALT activity, it is unlikely there would be unwanted consequences of this action; the other two causes are possible.

Many side effects are relatively mild in most people or can be mitigated by other drugs. However, drugs which cause lasting harm due to toxicity can normally only be used for limited periods and are rarely used in children. There are potential effects on development or lifelong harm. A PC for galactosemia is likely to be started in the first weeks of life and continued throughout the life of the patient. Therefore, cumulative toxicity could be an issue as lipophilic drugs will build up in adipose tissue over time. Since clinical trials are carried out over a limited period, not all toxicities will be apparent at the time of publishing and will only become apparent after years of follow up.

There is a lack of data on the galactosemic phenotype in older patients; most studies focus on children or adults under 30 years old. Therefore, it may be difficult to determine whether symptoms are due to disease phenotype or potential drug toxicities. In this population, there is also a greater chance of comorbidities and interactions with the drugs used to treat these.

### 5.4. Costs and Returns on Investments

Drug discovery is expensive and is normally financed by the profits derived from the sale of existing drugs. This means that pharmaceutical companies may be reluctant to invest in drugs to treat rare diseases such as galactosemia. This problem can be partly alleviated by governments or charities sharing costs and risks. Any PC for galactosemia is likely to be granted orphan drug status. The definition of orphan drug differs depending on the jurisdiction, but it is largely dependent on the disease being rare and therefore a drug having little profitability, but which would meet a public health need. Orphan drug status can have benefits such as tax incentives, direct government subsidiary, increased patent time or a streamlined approval process [89].

Nevertheless, return on investment for pharmaceutical companies is likely to occur over the longer term (50+ years), if at all. This will require long-term strategies by the pharmaceutical industry which are likely to extend beyond the complete turnover of their boards of directors. Initially, the likely lack of competition may encourage companies to develop these kinds of drugs. However, this lack of competition may mean other companies do not seek to develop rival drugs with improved efficacy.

## 6. Conclusions

As in many other rare, inherited metabolic diseases, galactosemias lack adequate treatments or cures. Future research must focus on identifying safe and efficient new treatment options for these patients. Despite the significant genetic diversity found in all types of galactosemias, most mutations impact the stability of the corresponding protein or its ability to fold properly in vivo. Consequently, the development of PCs has emerged as a plausible approach for future therapies in galactosemias. Easy recombinant production and in vitro characterization of wild type and mutant variant proteins, the existence of simple and easy to handle eukaryotic expression systems (e.g., budding yeast), and the availability of high resolution crystal structures will hopefully allow the identification of potential PCs for these diseases, using different types of in vitro and in silico approaches.

An effective PC for galactosemia would be a significant improvement to the current treatment mantra of maintaining a galactose-free diet. If started soon after birth, using a PC to restore some enzyme function could be life-changing for patients, improving both their morbidity and mortality by helping to avoid harmful complications such as liver dysfunction and cataracts.

## Data Availability

Data sharing not applicable.
